# Two new species of the genus *Macrothele* Ausserer, 1871 (Araneae, Macrothelidae) from China

**DOI:** 10.3897/BDJ.10.e90967

**Published:** 2022-11-01

**Authors:** Yaying Wu, Zhimin Li, Yan Yang, Zizhong Yang

**Affiliations:** 1 Yunnan Provincial Key Laboratory of Entomological Biopharmaceutical R&D, Dali University, Yunnan Dali, China Yunnan Provincial Key Laboratory of Entomological Biopharmaceutical R&D, Dali University Yunnan Dali China; 2 National-Local Joint Engineering Research Center of Entomoceutics, Dali University, Yunnan Dali, China National-Local Joint Engineering Research Center of Entomoceutics, Dali University Yunnan Dali China; 3 Administration of Nangunhe National Nature Reserve, Yunnan, Cangyuan, China Administration of Nangunhe National Nature Reserve, Yunnan Cangyuan China; 4 Yunnan Provincial Science and Technology, Yunnan Kunming, China Yunnan Provincial Science and Technology Yunnan Kunming China

**Keywords:** mygalomorph, Macrothelidae, taxonomy, morphology, new species

## Abstract

**Background:**

The family Macrothelidae Simon, 1892 belongs to the infraorder Mygalomorphae, currently contains two genera and 47 described species, from South Europe, South, and East Southeast Asia, Central, West, and North Africa.

**New information:**

Two new species of the funnel-web spider genus *Macrothele* Ausserer, 1871 from Yunnan Province, China are described: *Macrothelewashanensis* Wu & Yang, sp. n. (♂♀), and *M.wuliangensis* Wu & Yang, sp. n. (♂♀). Detailed descriptions, diagnostic illustrations and distribution map are provided. All specimens are deposited in the Institute of Entomoceutics Research, Dali University (DUIER).

## Introduction

The spider family Macrothelidae Simon 1892 is an important spider group in the infraorder Mygalomorphae. They usually build funnel webs using crevices and cavities in slopes, occasionally build web in surface deciduous layers. So far, the family has 47 species of two genera reported worldwide (World Spider Catalog 2022), of which 29 species are known from China (Pocock 1901, Saitô 1933, Hu and Li 1986, Shimojana and Haupt 1998, Song et al. 1999, Zhu et al. 2000, Zhu and Song 2000, Xu and Yin 2001, Xu et al. 2002, Li and Zha 2013, Shi et al. 2018, Yang et al. 2018, Yang et al. 2019, Wang et al. 2019, Tang et al. 2020, Chen et al. 2020, Lin et al. 2021, Tang et al. 2022).

We are carrying out a systematic investigation on the Chinese fauna of Macrothelidae and have collected a lot of specimens from Yunnan Province. During this study, two new species have been discovered and described here: *Macrothelewashanensis* Wu & Yang, sp. n. and *M.wuliangensis* Wu & Yang, sp. n.

## Materials and methods

Specimens were examined and measured with Olympus SZX16 and Leica M205A stereo-microscopes and an Olympus CX33 compound microscope. All specimens examined were preserved in 80% ethanol. The left male palps were examined after dissection and removal from the specimens, and the female genitalia were treated in 10% NaOH for 24 hours to dissolve tissue and examine the vulvae. The distribution map was produced by ArcMap software (version 10.8).

All type specimens are deposited in Institute of Entomoceutics Research, Dali University (DUIER). All measurements are in millimeters. Palp and leg measurements are given as: palp total length (femur, patella + tibia, tarsus); leg total length (femur, patella + tibia, metatarsus, tarsus). Abbreviations used are: ALE = anterior lateral eyes; AME = anterior median eyes; PLE = posterior lateral eyes; PME = posterior median eyes; PMS = posterior median spinnerets; PLS = posterior lateral spinnerets; basal haematodocha = BH; embolus = E ; CD = copulatory ducts；T = terminus of receptacula.

## Taxon treatments

### Macrothele washanensis

Wu & Yang sp. n.

01B89F2B-E7E9-590F-B009-23B2E98C9F5D

259FAB90-0670-4940-9A06-49B730786D34

#### Materials

- **Type status:** Holotype. **Occurrence:** recordedBy: Zizhong Yang, Yongming You and Jiasen Wei; individualCount: 1; sex: male; lifeStage: adult; occurrenceID: D04682CE-CED5-56AC-A410-7702384D544D; **Taxon:** scientificName: *Macrothelewashanensis*; order: Araneae; family: Macrothelidae; genus: Macrothele; **Location:** country: China; stateProvince: Yunnan; county: Cangyuan county; municipality: Lincang; verbatimLatitude: 23°14′5′′N; verbatimLongitude: 99°17′48′′E; **Event:** year: 2021; month: 5; day: 10; **Record Level:** institutionCode: DUIER-MWa11
- **Type status:** Paratype. **Occurrence:** recordedBy: Zizhong Yang, Yongming You and Jiasen Wei; individualCount: 5; sex: 2 males, 3 females; lifeStage: adult; occurrenceID: 591EC1BF-750A-582E-9048-CB6B879AA1AE; **Taxon:** scientificName: *Macrothelewashanensis*; order: Araneae; family: Macrothelidae; genus: Macrothele; **Location:** country: China; stateProvince: Yunnan; county: Cangyuan county; municipality: Lincang; verbatimLatitude: 23°14′5′′N; verbatimLongitude: 99°17′48′′E; **Event:** year: 2021; month: 5; day: 10; **Record Level:** institutionCode: DUIER-MWa21-MWa25
- **Type status:** Other material. **Occurrence:** recordedBy: Zizhong Yang, Chenggong Li and Wenjie Zhang; individualCount: 6; sex: 4 males, 2 females; lifeStage: 2 males juveniles, 2 females juveniles; occurrenceID: 1CB3C2AB-A8F3-597A-B327-116255DB7C73; **Taxon:** scientificName: *Macrothelewashanensis*; order: Araneae; family: Macrothelidae; genus: Macrothele; **Location:** country: China; stateProvince: Yunnan; county: Gengma county; municipality: Lincang; locality: Mengjian Town; verbatimLatitude: 23°43'0.772″N; verbatimLongitude: 99°20'14.784"E; **Event:** year: 2017; month: 9; day: 15; **Record Level:** institutionCode: DUIER-MWa26-MWa211
- **Type status:** Other material. **Occurrence:** recordedBy: Zizhong Yang, Chenggong Li and Wenjie Zhang; individualCount: 23; sex: females; lifeStage: 22 females juveniles; occurrenceID: C8434D9A-AFE5-5421-BEB6-35D4F206CE28; **Taxon:** scientificName: *Macrothelewashanensis*; order: Araneae; family: Macrothelidae; genus: Macrothele; **Location:** country: China; stateProvince: Yunnan; county: Gengma county; municipality: Lincang; locality: Lixin village; verbatimLatitude: 23º15′31.90″N; verbatimLongitude: 99º17′22.45″E; **Event:** year: 2018; month: 7; day: 25; **Record Level:** institutionCode: DUIER-MWa212-MWa234

#### Description

**Figure 1.**
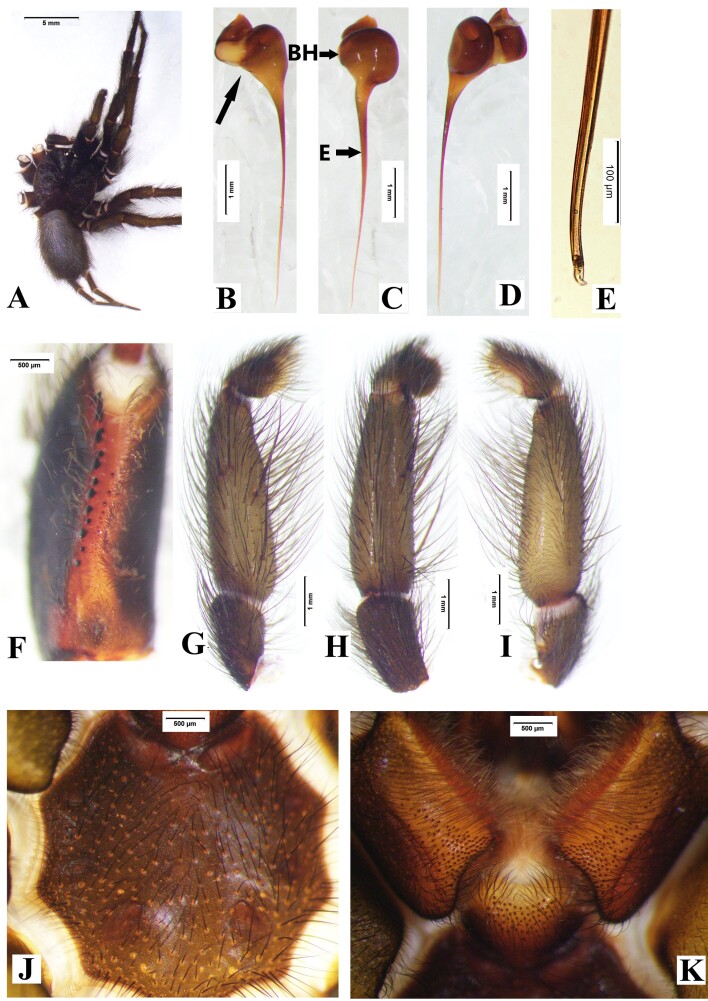
*Macrothelewashanensis* sp. n., male holotype. **A.** Male body, dorsal view; **B.** Embolus, prolateral view; **C.** Same, ventral view; **D.** Same, retrolateral view; **E.** Same, the end; **F.** Left chelicerae, ventral view; **G.** Left palp tibia, prolateral view; **H.** Same, dorsal view; **I.** Same, retrolateral view; **J.** Sternum; **K.** Maxillae and labium.

**Male** (holotype, DUIER-MWa11, Fig. 1). Total length 24.56: cephalothorax 10.89 long, 6.98 wide; opisthosoma 8.14 long, 5.64 wide. Carapace dark, gray hairs. Fovea concave. Both eye rows recurved. Eye sizes and inter-distances: AME 0.55, ALE 0.64, PME 0.48, PLE 0.49; ALE-AME 0.14, AME-AME 0.17, ALE-PLE 0.18, PLE-PME 0.07, PME-PME 0.88. Eye area 1.11 long, 2.21 wide. Chelicerae black, with 15 stout promarginal teeth, 13 small retromarginal teeth, and 15 tiny teeth within fang furrow (Fig. 1F). Labium and maxillae chestnut, with grey bristles and patch of cuspules on anterior half part (Fig. 1K); maxillae area length 2.58, and cuspules area length 1.45. Sternum with three pairs of sigilla (Fig. 1J). Palp: tibia grey, 4.34 long, with four long prolateral spines (Fig. 1G), palpal trochanter without lyral spines; embolus 4.76 long, apex curved and hook shaped (Fig. 1B-E).

**Figure 2.**
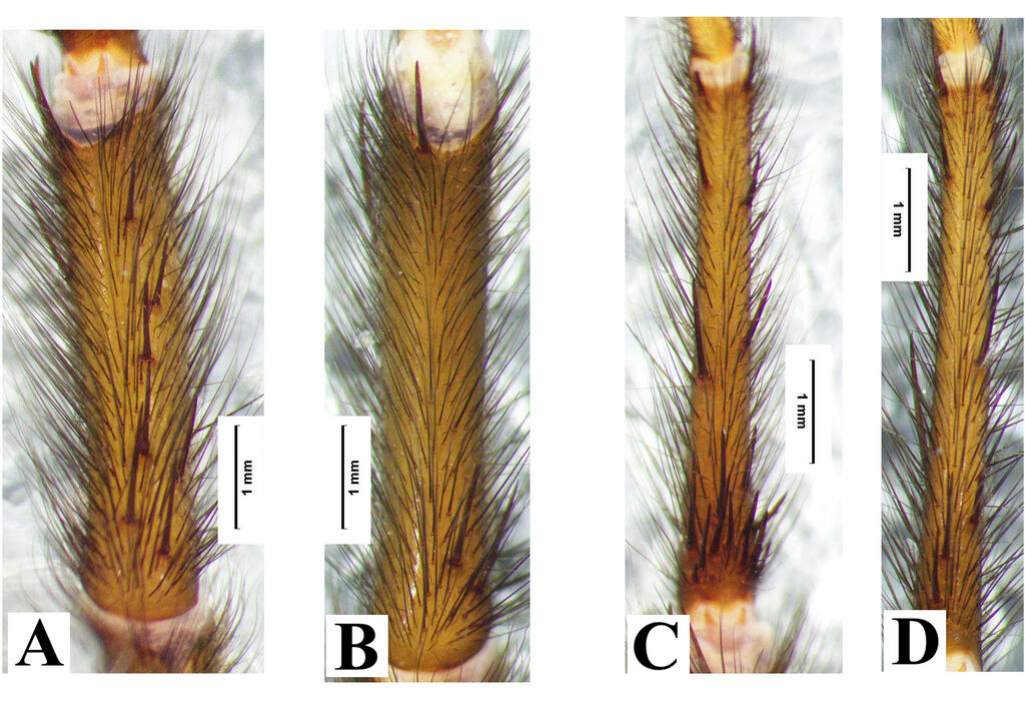
*Macrothelewashanensis* sp. n., male holotype, left leg. **A.** Tibia I, ventral view; **B.** Metatarsus I, ventral view; **C.** Tibia II, ventral view; **D.** Metatarsus II, ventral view.

**Figure 3.**
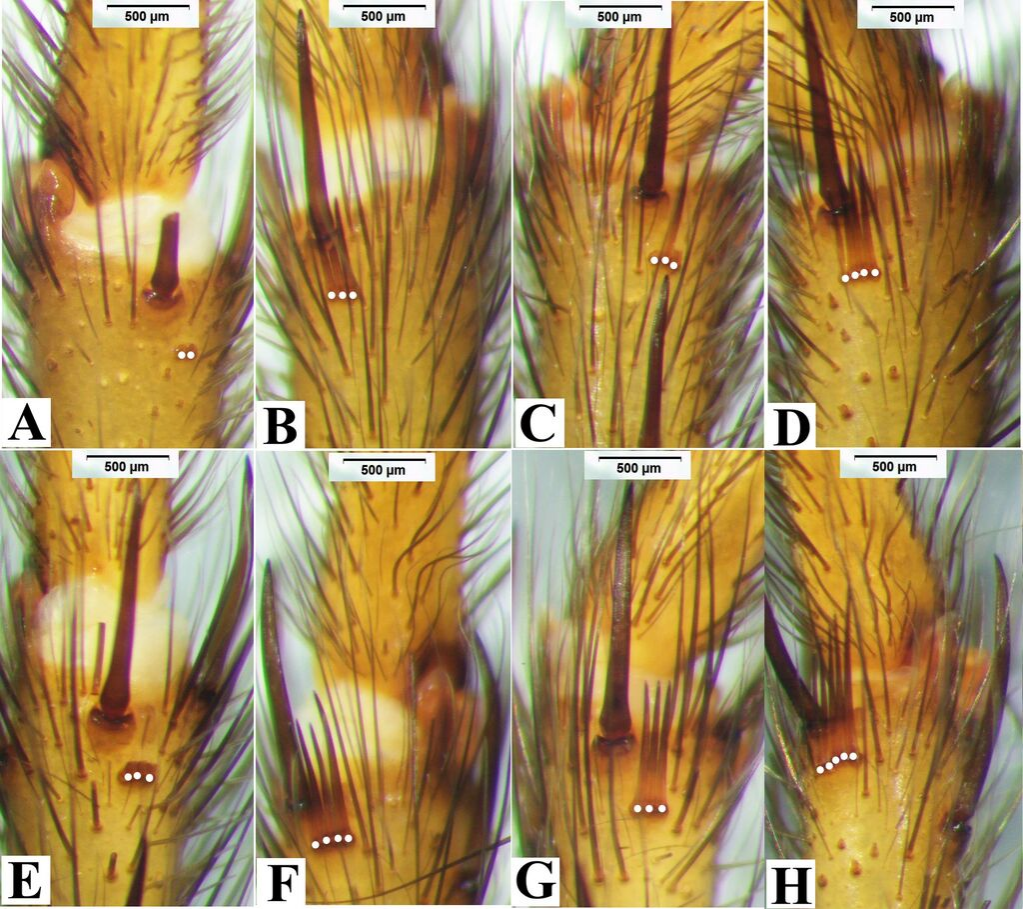
*Macrothelewashanensis* sp. n., male holotype, comb-like organs. **A.** Metatarsus I, prolateral comb-like organs; **B.** Same, retrolateral comb-like organs; **C.** Metatarsus II, prolateral comb-like organs; **D.** Same, retrolateral comb-like organs; **E.** Metatarsus III, prolateral comb-like organs; **F.** Same, retrolateral comb-like organs; **G.** Metatarsus IV, prolateral comb-like organs; **H.** Same, retrolateral comb-like organs.

Leg spines. I: femur with 1 prolateral near base; patella 2 prolateral distally; tibia 2 prolateral, 2 retrolateral, 9 ventral (Fig. 2A); metatarsus with 19 ventral spines (Fig. 2B), and two comb-like organs near distad, among them, prolateral with 2 spines, retrolateral with 3 spines (Fig. 3A-B); tarsus with 3 prolateral and 3 retrolateral. II: femur 1 prolateral near distad; patella 2 prolateral; tibia 1 prolateral, and 3 ventral, 1 spine near distad, 2 near basal (Fig. 2C); metatarsus with 1 prolateral, 10 ventral (Fig. 2D), two comb-like organs near distad, among them, prolateral with 3 spines, retrolateral with 4 spines (Fig. 3C-D); tarsus with 6 prolateral, 6 retrolateral. III: femur 1 prolateral and 1 retrolateral; patella 2 prolateral, 1 retrolateral; tibia 3 prolateral, 2 retrolateral, and 3 ventral; metatarsus 2 ventral, 4 prolateral, 3 retrolateral, two comb-like organs near distad, among them, prolateral with 3 spines, retrolateral with 4 spines (Fig. 3E-F); tarsus with 4 prolateral, 5 retrolateral. IV: femur 1 prolateral and retrolateral near distal end; patella with 2 prolateral, 1 retrolateral; tibia with 2 prolateral, 1 retrolateral, 3 ventral near distal end; metatarsus with 2 dorsal, 3 prolateral, 3 retrolateral, 7 ventral, and two comb-like organs near distad, among them, prolateral with 3 spines, retrolateral with 5 spines (Fig. 3G-H); tarsus with 7 prolateral, 6 retrolateral.

Measurements of palp and legs: palp: 9.63 (2.28, 6.12, 1.23); I: 22.79 (6.14, 7.66, 5.58, 3.41); II: 25.80 (6.72, 8.75, 6.84, 3.49); III: 24.69 (6.14, 7.89, 6.93, 3.73); IV: 29.75 (7.37, 9.35, 8.68, 4.35). Leg formula: 4231.

Abdomen gray brown, hairy. Spinnerets, PMS one segment, 1.87 long, 0.48 wide, PMS-PMS 0.78; PLS three segments, PLS 10.65 long (3.20, 3.19, 4.26).

**Figure 4.**
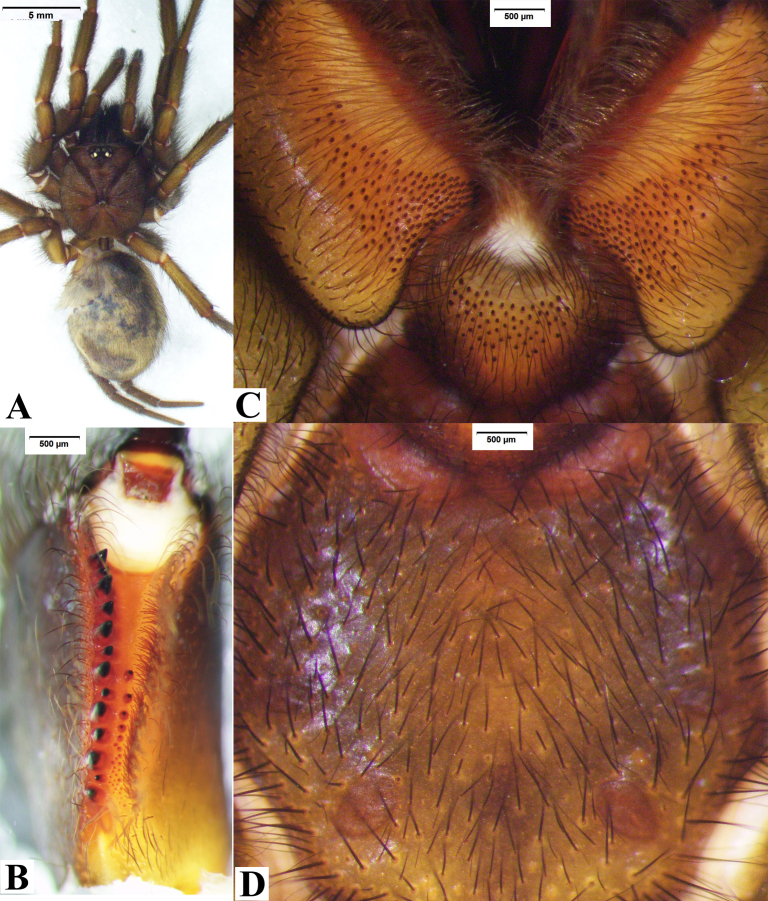
*Macrothelewashanensis* sp. n., female paratype. **A.** Female, body, dorsal view; **B.** Left chelicerae, ventral view; **C.** Maxillae and labium; **D.** Sternum.

**Female** (DUIER-MWa21, Fig. 4). Total length 28.39 : cephalothorax 11.67 long, 6.66 wide; opisthosoma 9.11 long, 6.54 wide. Eye sizes and inter-distances: AME 0.52, ALE 0.71, PME 0.50, PLE 0.51; AME-AME 0.26, ALE-AME0.24, ALE-PLE 0.12, PME-PME 0.91, PLE-PME 0. Eye area 1.13 long, 2.24 wide. Chelicerae brown, with 15 stout promarginal teeth, 12 small retromarginal teeth, and 13 tiny teeth within fang furrow (Fig. 4B). Labium and maxillae dark yellow, with dark brown bristles and patch of cuspules on anterior half part (Fig. 4C); maxillae area length 2.34, and cuspule area length 1.58. Sternum with three pairs of sigilla (Fig. 4D); palpal trochanter without lyral spines.

**Figure 5.**
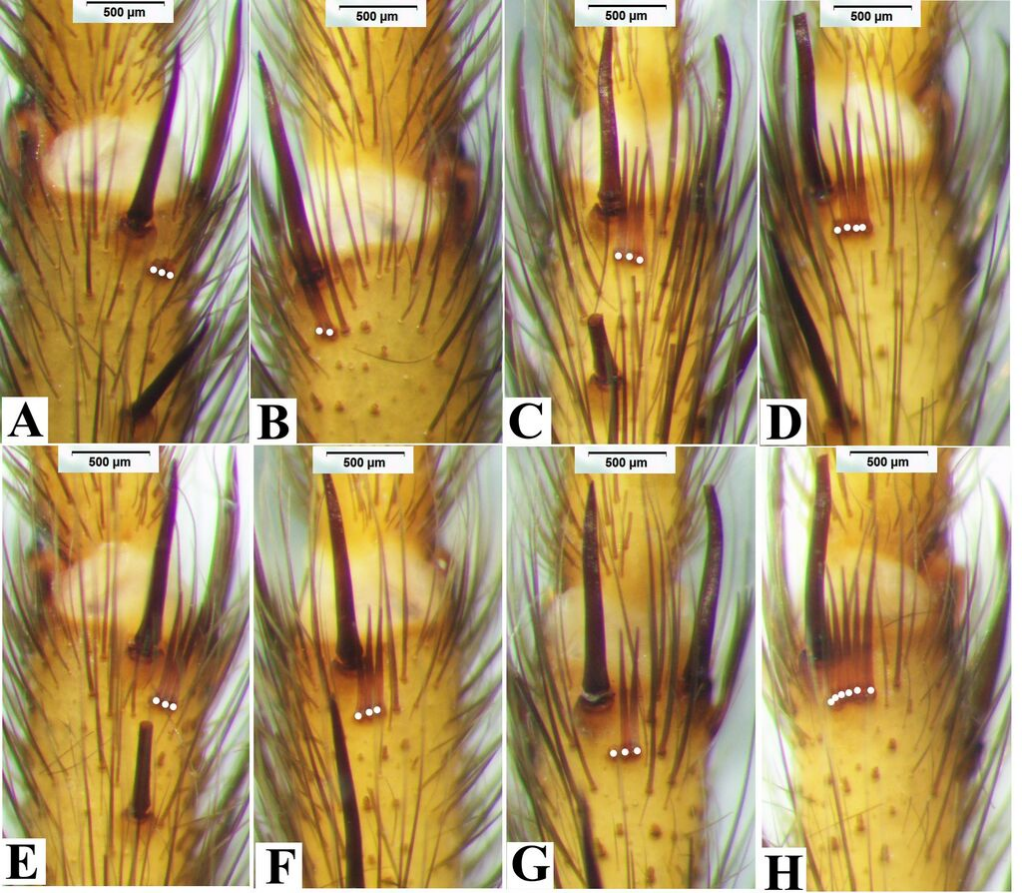
*Macrothelewashanensis* sp. n., female paratype. **A.** Metatarsus I, prolateral comb-like organs; **B.** Same, retrolateral comb-like organs; **C.** Metatarsus II, prolateral comb-like organs; **D.** Same, retrolateral comb-like organs; **E.** Metatarsus III, prolateral comb-like organs; **F.** Same, retrolateral comb-like organs; **G.** Metatarsus IV, prolateral comb-like organs; **H.** Same, retrolateral comb-like organs.

Leg spines. I: femur with 1 prolateral near distad; patella 1 prolateral; tibia with 1 prolateral; metatarsus with 7 ventral, and two comb-like organs near distad, prolateral with 3 spines, retrolateral with 2 spines (Fig. 5A-B); tarsus with 3 prolateral, 4 retrolateral. II: femur 1 prolateral near distad; patella 2 prolateral; tibia 1 prolateral, 2 ventral; metatarsus 1 prolateral, 6 retrolateral, and two comb-like organs near distad, among them, prolateral with 3 spines, retrolateral with 4 spines (Fig. 5C-D); tarsus with 5 prolateral, 3 retrolateral. III: femur with 1 dorsal; patella 2 prolateral and 1 retrolateral; tibia 3 prolateral, and 2 retrolateral, 3 ventral distally; metatarsus with 2 dorsal, 5 prolateral, 4 retrolateral, 6 ventral, and two comb-like organs near distad, among them, prolateral with 3 spines, retrolateral with 3 spines (Fig. 5E-F); tarsus with 5 prolateral, 6 retrolateral. IV: femur 1 prolateral near distad, 1 retrolateral; patella 2 prolateral, 1 retrolateral; tibia 3 prolateral, and 2 retrolateral, 3 ventral near distad; metatarsus with 2 dorsal, 3 prolateral, 4 retrolateral, 7 ventral, and two comb-like organs near distad, prolateral with 3 spines, retrolateral with 6 spines (Fig. 5G-H); tarsus with 9 prolateral, 7 retrolateral.

Measurements of palp and legs: palp: 12.07 (3.07, 4.80, 4.20); I: 20.65 (5.80, 7.47, 4.85, 2.53); II: 21.99 (5.81, 7.92, 5.33, 2.93); III: 22.24 (5.39, 7.25, 6.33, 3.27); IV: 25.37 (6.24, 8.35, 7.54, 3.24). Leg formula: 4321.

**Figure 6.**
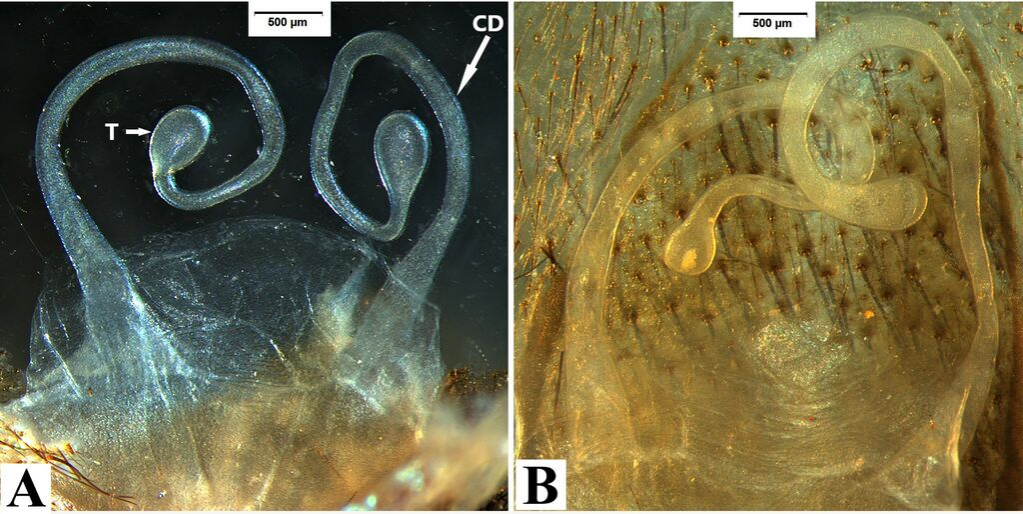
*Macrothelewashanensis* sp. n., female paratype. **A-B.** Genitalia, Cangyuan county.

Receptacula apically teardrop shaped, the ratio of the length of the T to the length of the CD is almost 1:6 (Fig. 6A-B). Spinnerets: PMS one segment, PMS 2.78 long, 0.58 wide, PMS-PMS 1.63; PLS three segments, PLS 11.11 long (2.91, 3.75, 4.45).

#### Diagnosis

Males of *Macrothelewashanensis* sp. n. resemble *M.arcuata* Tang, Zhao & Yang, 2020 by having similar bulb shape, but they can be distinguished by the BH no protrusion in prolateral view, embolus tapers from base to apex, and hook-shaped, the ratio of the length of the BH to the length of the E is almost 1 : 4 (Fig. 1B-E); the four tibial spines visible in prolateral view (Fig. 1G-I); tibia I with nine spines visible in ventral view, tibia II straight, with three ventral spines (Fig. 2) (vs tibia with three prolateral spines, and three ventral spines, embolus with visible protrusion, joint of embolus and bulb is strongly bent, embolus needle shaped, the ratio of the length of the BH to the length of the E is almost 1 : 5; tibia I with 26 spines, tibia II with retrolateral bend and 15 ventral spines in *M.arcuata*). Females of *M.washanensis* sp. n. can be differentiated from *M.arcuata* by the receptacula apically teardrop shaped, the ratio of the length of the T to the length of the CD is almost 1 : 6 (Fig. 6) (vs copulatory duct long, shape of the English letter “G”; receptacula apically oval, the ratio of the length of the T to the length of the CD is almost 1 : 8 in *M.arcuata*).

#### Etymology

The species epithet is a noun in apposition referring to the type locality.

#### Distribution

China, Yunnan Province (Cangyuan, Gengma) (Fig. 15)

#### Ecology

**Figure 7.**
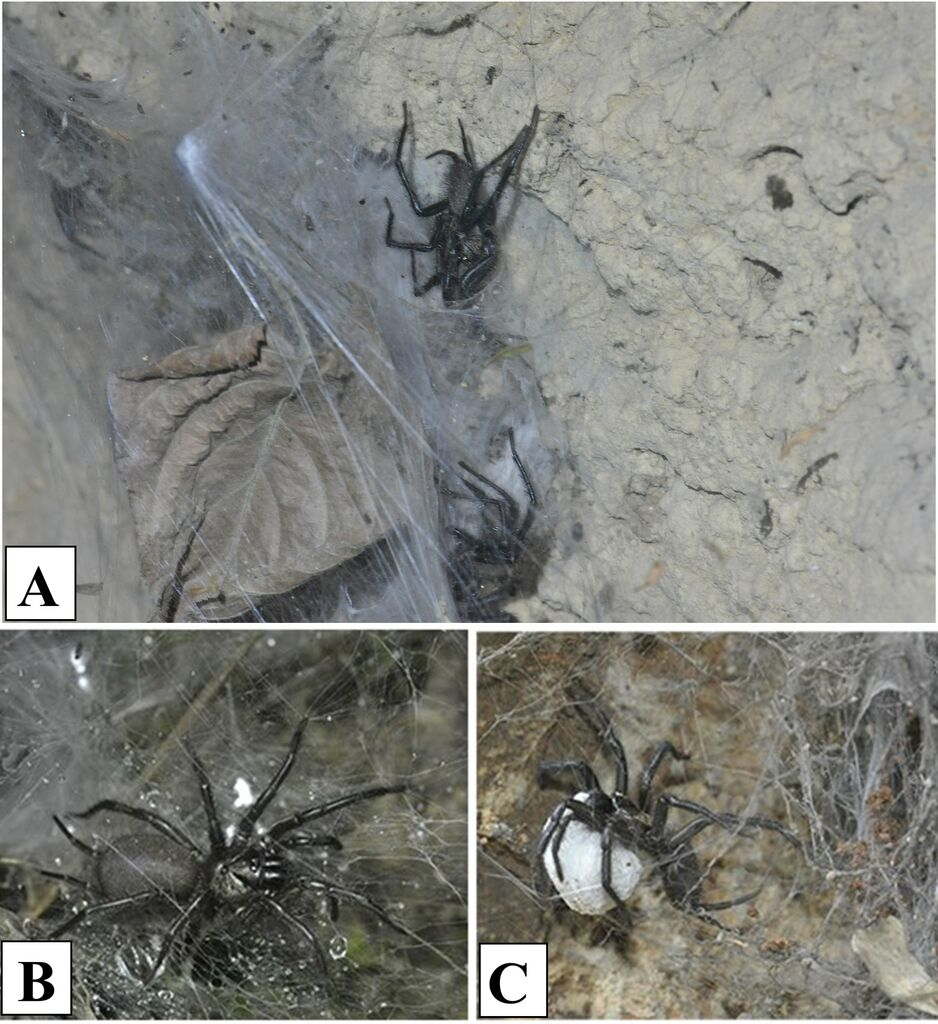
*Macrothelewashanensis* sp. n. **A.** Spider shelter and spiders; **B.** Living female; **C.** Female spider with eggs.

Spinning large funnel web on crevices. Female often stays in the entrance of funnel tube, when the sheet part of funnel web was hit by other animals, she quickly rush out, to catch the prey, or attack the enemy (Fig. 7B). If the male of the same species comes, releasing some chemical clue or sending vibration via the web, female accepted the clue and walk out for further communication and copulation (Fig. 7A). After some days of copulation, female will lay several dozens of eggs, packaged in a silk-sac, then carried it under her ventral side with cheliceral fang (Fig. 7C).

#### Variation

Male (holotype and two male paratypes, n = 3): total length 23.30-24.56; maxillary cuspules 220-269, labial cuspules 53-59. Female (paratypes, n = 3): total length 23.43-28.39; maxillary cuspules 260-297, labial cuspules 61-70.

### Macrothele wuliangensis

Wu & Yang sp. n.

0E37C13A-E435-5485-908D-E29F52ACEEA0

DA15CB9B-018A-42F1-A34B-28DE1240985A

#### Materials

- **Type status:** Holotype. **Occurrence:** recordedBy: Zizhong Yang; individualCount: 1; sex: male; lifeStage: adult; occurrenceID: C1EDAD90-9A3D-5884-A92F-7FE5C002F5FF; **Taxon:** scientificName: *Macrothelewuliangensis*; order: Araneae; family: Macrothelidae; genus: Macrothele; **Location:** country: China; stateProvince: Yunnan; county: Jingdong county; municipality: Puer; verbatimLatitude: 24°31ʹ38ʺN; verbatimLongitude: 100°47ʹ47ʺE; **Event:** year: 2019; month: 5; day: 28; **Record Level:** institutionCode: DUIER-MWl11
- **Type status:** Paratype. **Occurrence:** recordedBy: Dasong Yang, Yani Tang, Ying Wang and Lei Tao; individualCount: 18; sex: 2 males, 16 females; lifeStage: 10 adults, 8 females juveniles; occurrenceID: 751DC632-5A7E-5A49-87A8-7BF2EF11204E; **Taxon:** scientificName: *Macrothelewuliangensis*; order: Araneae; family: Macrothelidae; genus: Macrothele; **Location:** country: China; stateProvince: Yunnan; county: Jingdong county; municipality: Puer; verbatimLatitude: 24°31ʹ38ʺN; verbatimLongitude: 100°47ʹ47ʺE; **Event:** year: 2019; month: 5; day: 28; **Record Level:** institutionCode: DUIER-MWl21-MWl218
- **Type status:** Other material. **Occurrence:** recordedBy: Zizhong Yang and Ping Feng; individualCount: 4; sex: 1male, 3 females; lifeStage: adult; occurrenceID: 7AFC9F9E-108B-5934-A860-66DB903D504F; **Taxon:** scientificName: *Macrothelewuliangensis*; order: Araneae; family: Macrothelidae; genus: Macrothele; **Location:** country: China; stateProvince: Yunnan; county: Zhenyuan county; municipality: Puer; locality: Bollie River; verbatimLatitude: 23°52.536′N; verbatimLongitude: 101°08.416′E; **Event:** year: 2010; month: 7; day: 22; **Record Level:** institutionCode: DUIER-MWl219-MWl222
- **Type status:** Other material. **Occurrence:** recordedBy: Zizhong Yang and Chenghong Li; individualCount: 4; sex: 4 females; lifeStage: 3 juveniles; occurrenceID: 2CC9FE88-81E8-5AF1-A744-DEBCB84A7DA8; **Taxon:** scientificName: *Macrothelewuliangensis* sp. n.; order: Araneae; family: Macrothelidae; genus: Macrothele; **Location:** country: China; stateProvince: Yunnan; county: Zhenyuan county; municipality: Puer; locality: Gucheng Town; verbatimLatitude: 23º29’52.5″N; verbatimLongitude: 101º10’33.8″E; **Event:** year: 2017; month: 3; day: 7; **Record Level:** institutionCode: DUIER-MWl223-MWl226
- **Type status:** Other material. **Occurrence:** recordedBy: Zizhong Yang and Wanping Li and Shengshuai Liu; individualCount: 13; sex: 13 females; lifeStage: 8 adults, 5 juveniles; occurrenceID: BF23457D-A6A1-5987-B572-D3BF4C5DF46E; **Taxon:** scientificName: *Macrothelewuliangensis*; order: Araneae; family: Macrothelidae; genus: Macrothele; **Location:** country: China; stateProvince: Yunnan; county: Jingdong county; municipality: Puer; locality: Jingping Town; verbatimLatitude: 24º34’52.4″N; verbatimLongitude: 100º46’35.9″E; **Event:** year: 2018; month: 9; day: 14; **Record Level:** institutionCode: DUIER-MWl236-MWl248

#### Description

**Figure 8.**
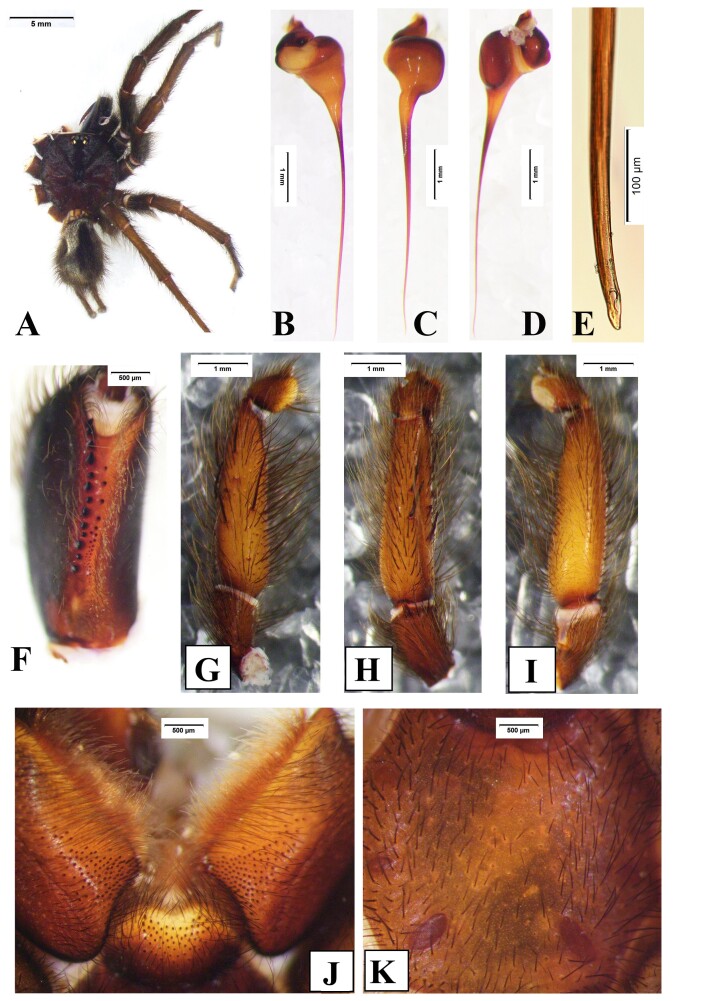
*Macrothelewuliangensis* sp. n., male holotype. **A.** Male body, dorsal view; **B.** Embolus, prolateral view; **C.** Same, ventral view; **D.** Same, retrolateral view; **E.** Same, the end; **F.** Left chelicerae. ventral view; **G.** Left palp tibia, prolateral view; **H.** Same, dorsal view; **I.** Same, retrolateral view; **J.** Sternum; **K**. Maxillae and labium.

**Male** (**holotype**, DUIER-MWl11, Fig. 8). Total Length 22.52: cephalothorax 11.43 long, 7.49 wide; opisthosoma 8.49 long, 4.97 wide. Carapace dark chestnut, gray hairy. Fovea concave. Both eye rows recurved. Eye sizes and inter-distances: AME 0.47, ALE 0.62, PME0.45, PLE 0.50; ALE-AME 0.15, AME-AME 0.31, ALE-PLE 0.22, PLE-PME 0.05, PME-PME 0.95. Eye area 1.15 long, 2.28 wide. Chelicerae dark, with 13 stout promarginal teeth, 15 small retromarginal teeth, 17 tiny teeth with fang furrow (Fig. 8F). Labium and maxillae chestnut, with dark brown bristles and patch of cuspules on anterior half part (Fig. 8J); maxillae area length 3.30, cuspules area length 1.48. Sternum chestnut, and with three pairs of sigilla (Fig. 8K). Palp: tibia yellow, 4.34 long，with one long and two stout prolateral spines, two stout dorsal spines (Fig. 8G-I), palpal trochanter without lyral spines; embolus terminal end curved, 4.76 long. (Fig. 8E)

**Figure 9.**
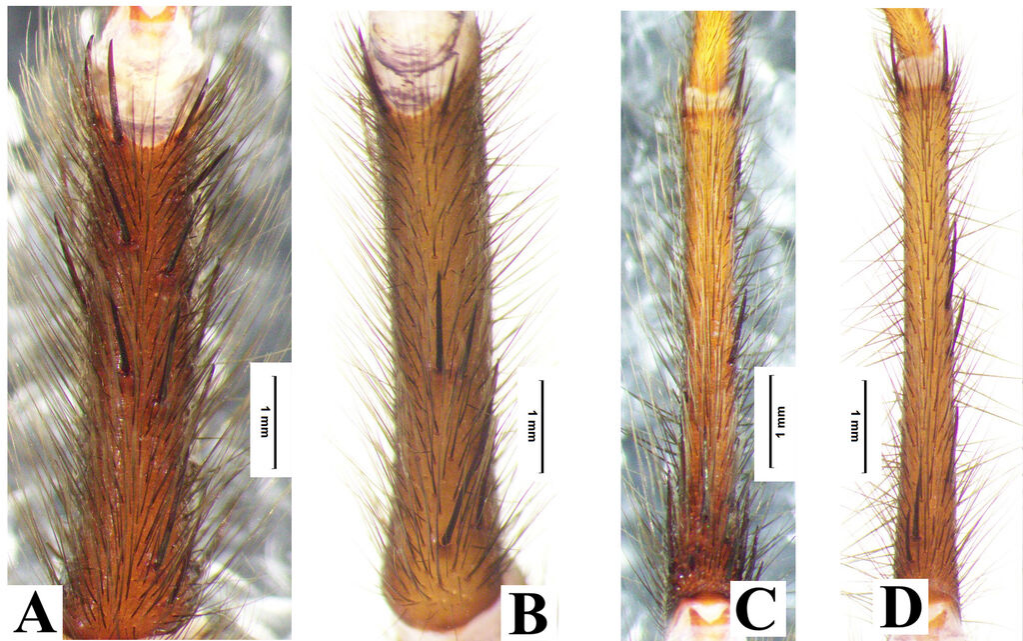
*Macrothelewuliangensis* sp. n., male holotype, left leg. **A.** Tibia I, ventral view; **B.** Metatarsus I, ventral view; **C.** Tibia II, ventral view; **D.** Metatarsus II, ventral view.

**Figure 10.**
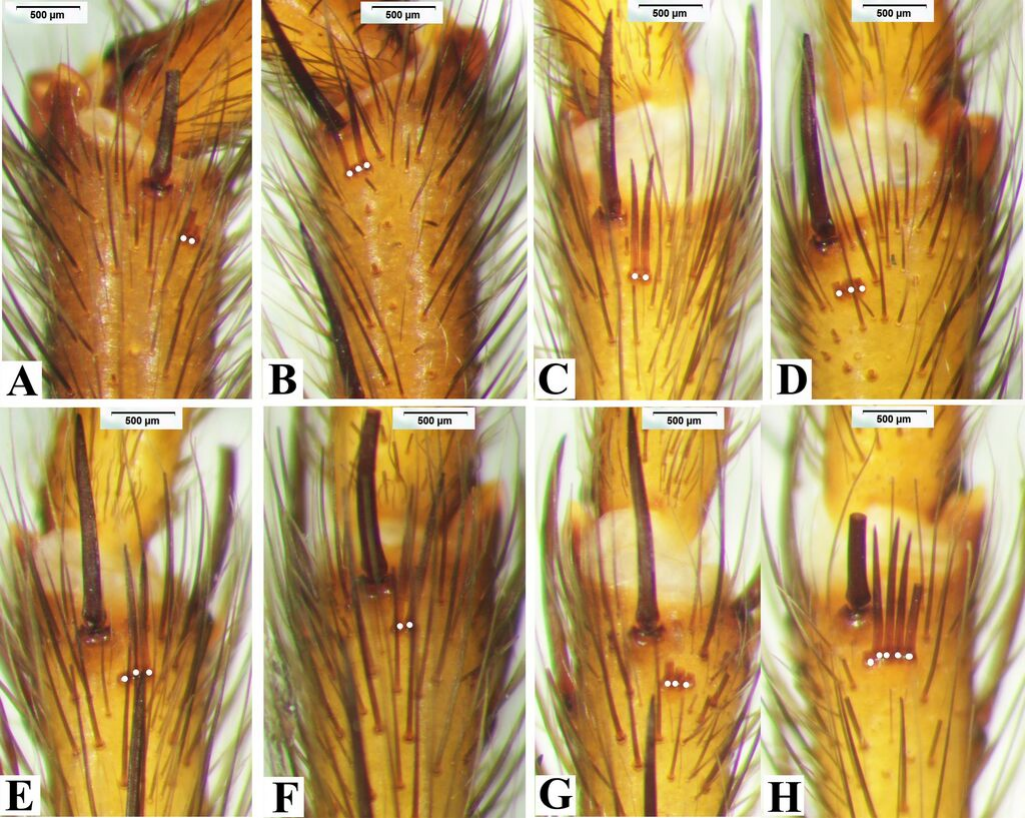
*Macrothelewuliangensis* sp. n., male holotype. **A.** Metatarsus I, prolateral comb-like organs; **B.** Same, retrolateral comb-like organs; **C.** Metatarsus II, prolateral comb-like organs; **D.** Same, retrolateral comb-like organs; **E.** Metatarsus III, prolateral comb-like organs; **F.** Same, retrolateral comb-like organs; **G.** Metatarsus IV, prolateral comb-like organs; **H.** Same, retrolateral comb-like organs.

Leg spines. I: femur with 1 dorsal near distad; tibia 2 prolateral, 3 retrolateral, 10 ventral (Fig. 9A) metatarsus with 16 ventral, 12 ventral on near based (Fig. 9B), and two comb-like organs near distad, among them, prolateral with 2 spines, retrolateral with 3 spines (Fig. 10A-B); tarsus 3 prolateral, 4 retrolateral. II: femur with 2 dorsal; patella 2 prolateral; tibia 1 prolateral, 7 ventral (Fig. 9C); metatarsus with 1 prolateral, 7 ventral (Fig. 9D), and two comb-like organs near distad, prolateral with 2 spines, retrolateral with 3 spines (Fig. 10C-D); tarsus 4 prolateral, 4 retrolateral. III: patella 2 prolateral, 1 retrolateral; tibia 3 prolateral, 1 retrolateral, 4 ventral; metatarsus 2 dorsal, 4 prolateral, 2 retrolateral, 7 ventral, and two comb-like organs near distad, among them, prolateral with 3 spines, retrolateral with 2 spines (Fig. 10E-F); tarsus with 7 prolateral, 5 retrolateral. IV: femur with 1 dorsal near basad; patella 2 prolateral, 1 retrolateral; tibia 2 prolateral, 2 retrolateral, 4 ventral; metatarsus 1 dorsal, 4 prolateral, 3 retrolateral, 7 ventral, and two comb-like organs near distad, among them, prolateral with 3 spines, retrolateral with 5 spines (Fig. 10G-H); tarsus with 7 prolateral, 7 retrolateral.

Measurements of palp and legs: palp: 12.30 (5.07, 6.22, 1.01); I: 22.44 (6.04, 7.81, 5.82, 2.77); II: 23.40 (6.09, 7.67, 6.29, 3.35); III: 22.77 (5.99, 7.38, 6.06, 3.34); IV: 27.76 (6.99, 8.75, 8.44, 3.58). Leg formula: 4231.

Abdomen black, hairy. Spinnerets: PMS one segment, 1.84 long, 0.45 wide, PMS-PMS 0.86; PLS three segments, PLS 10.35 long (3.29, 3.55, 3.51).

**Figure 11.**
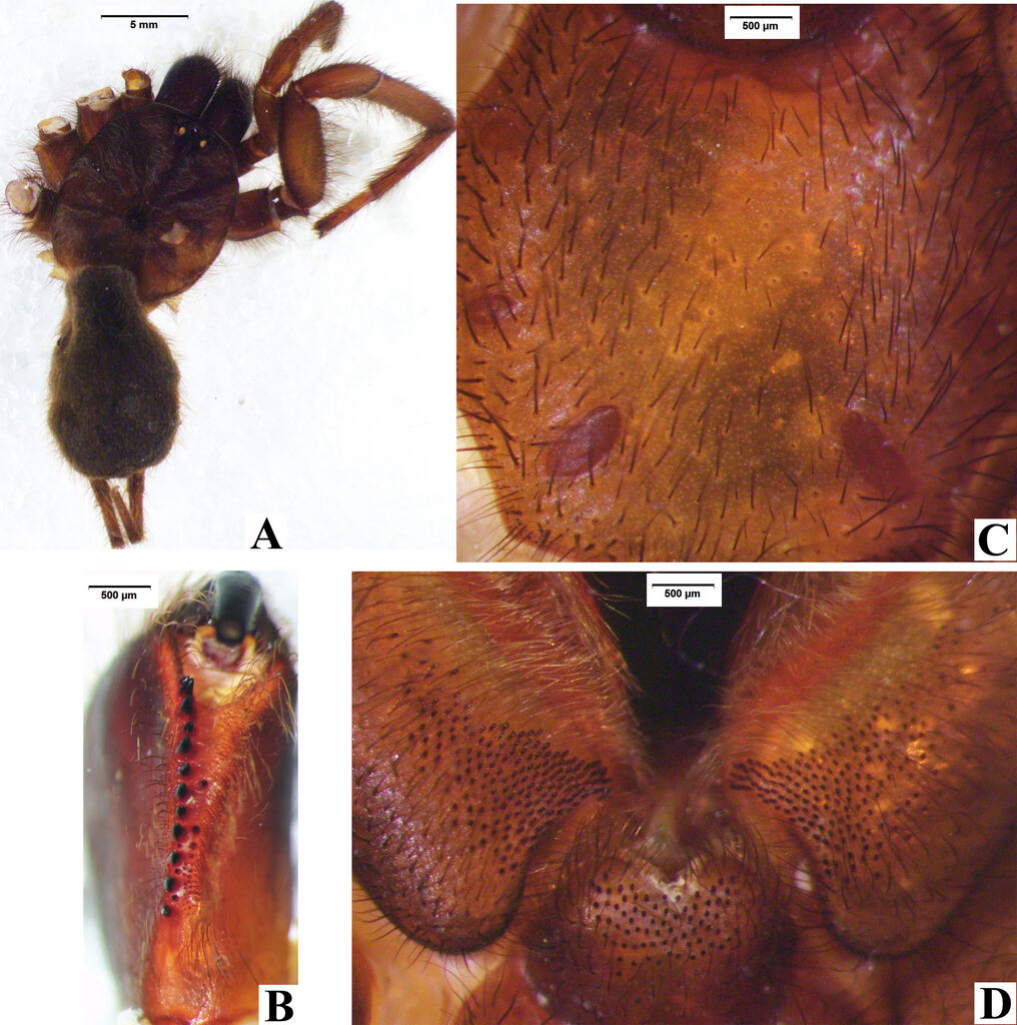
*Macrothelewuliangensis* sp. n., female paratype. **A.** Female, body, dorsal view; **B.** Left chelicerae, ventral view; **C.** Maxillae and labium; **D.** Sternum.

**Female** (DUIER-MWl21, Fig. 11). Total length 26.86: cephalothorax 8.95 long, 6.77 wide; opisthosoma 8.92 long, 5.18 wide. Eye sizes and inter-distances: AME 0.58, ALE 0.70, PME 0.50, PLE 0.56; AME-AME 0.17, ALE-AME 0.13, ALE- PLE 0.16, PLE-PME 0.03, PME-PME 0.99. Eye area 1.20 long, 2.50 wide. Chelicerae chestnut, with 15 stout promargin teeth, 12 small retromarginal teeth, 10 tiny teeth with fang furrow (Fig. 11B). Labium and maxillae lightly yellow, with dark brown bristles and patch of cuspules on anterior half part (Fig. 11C); maxillae area length 3.49, cuspules area length 2.15. Sternum yellow, with three pairs of sigilla (Fig. 11D); palpal trochanter without lyral spines.

**Figure 12.**
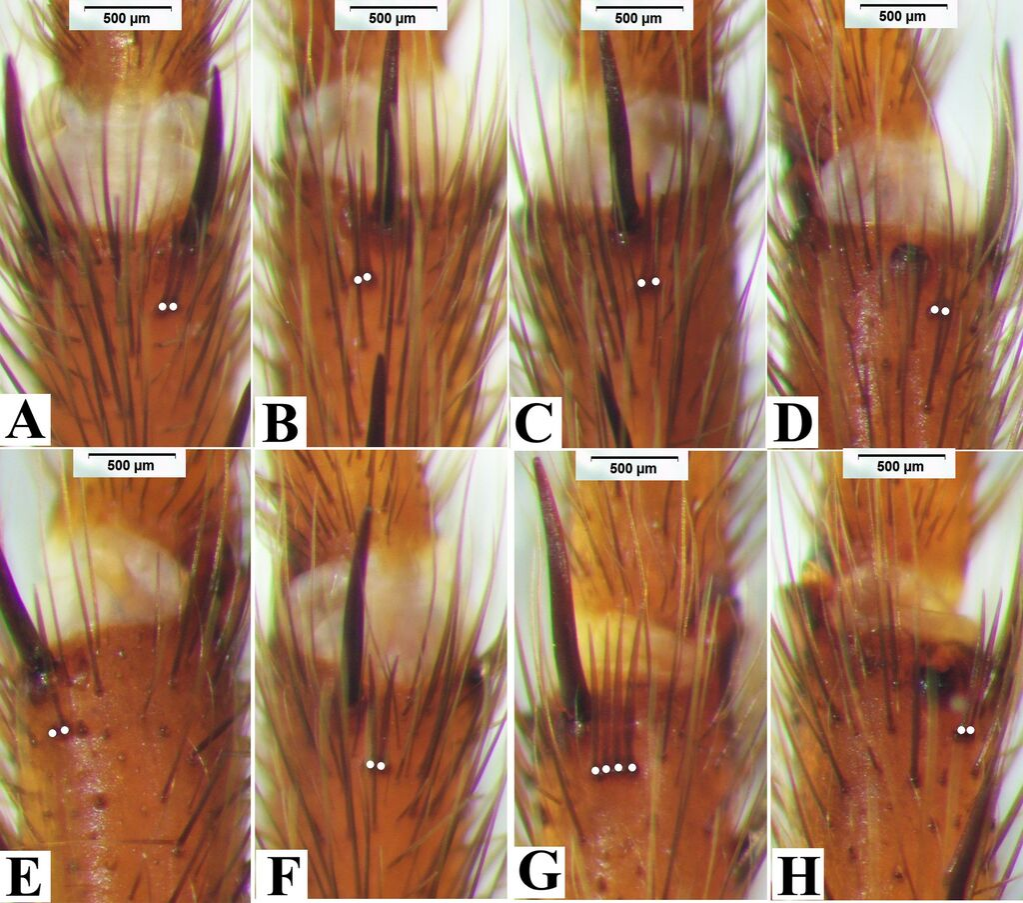
*Macrothelewuliangensis* sp. n., female paratype. **A.** Metatarsus I, ventral comb-like organs; **B.** Same, prolateral comb-like organs; **C.** Metatarsus II, retrolateral comb-like organs; **D.** Same, prolateral comb-like organs; **E.** Metatarsus III, retrolateral comb-like organs; **F.** Same, prolateral comb-like organs; **G.** Metatarsus IV, retrolateral comb-like organs; **H.** Same, prolateral comb-like organs.

Leg spines. I: patella with 1 prolateral; tibia 2 ventral; metatarsus 6 ventral, and two comb-like organs near distad, ventral with 2 spines, prolateral with 2 spines (Fig. 12A-B); tarsus 6 prolateral, 5 retrolateral. II: patella 1 prolateral; tibia 1 prolateral, 2 ventral; metatarsus 1 prolateral, 7 ventral, and two comb-like organs near distad, retrolateral with 2 spines, prolateral with 2 spines (Fig. 12C-D); tarsus 5 prolateral spines, 5 retrolateral. III: patella 2 prolateral, 1 retrolateral; tibia 4 prolateral, 2 retrolateral, 4 ventral distally; metatarsus 3 dorsal, 5 prolateral, 3 retrolateral, 8 ventral, and two comb-like organs near distad, among them, retrolateral with 2 spines, prolateral with 2 spines (Fig. 12E-F); tarsus 8 prolateral, 7 retrolateral. IV: patella 1 prolateral near distally, 1 retrolateral; tibia 2 prolateral, 2 retrolateral, 2 ventral distal; metatarsus 2 dorsal, 4 prolateral, 5 retrolateral, 8 ventral, and two comb-like organs near distad, among them, retrolateral with 4 spines, prolateral with 2 spines (Fig. 12G-H); tarsus with 9 prolateral, 8 retrolateral.

Measurements of palp and legs: palp: 13.52 (4.54, 4.91, 4.07); I: 18.86 (5.46, 6.58, 4.19, 2.63); II: 21.53 (6.77, 7.25, 4.86, 2.65); III: 23.00 (6.55, 8.33, 5.13, 2.99); IV: 23.33 (5.78, 8.51, 6.28, 2.76). Leg formula: 4321.

**Figure 13.**
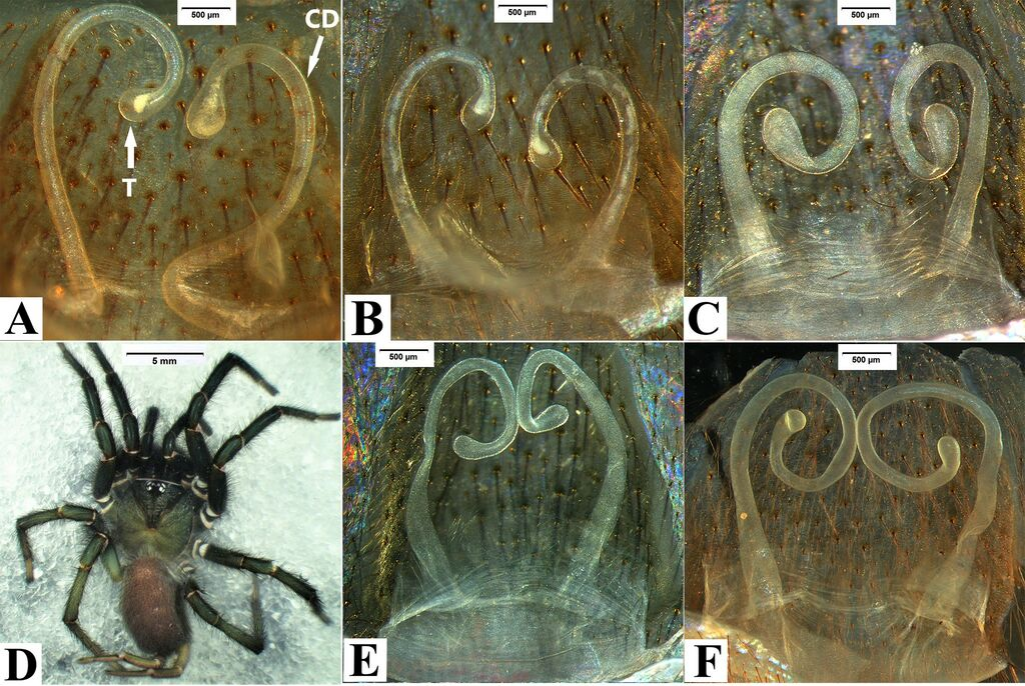
*M.wuliangensis* sp. n. **A.** Genitalia, paratype, Jingdong county; **B.** Same, Zhenyuan county Bollie River; **C.** Same, Jingdong county; **D.** Jingping Town, juvenile female; **E.** Same, juvenile genitalia; **F.** Zhenyuan county Bollie River, juvenile genitalia.

Apically teardrop shaped receptacula bent inwards apically, the ratio of the length of the T to the length of the CD is almost 1 : 5 (Fig. 13). Spinnerets: PMS one segment, 1.94 long, 0.59 wide, PMS-PMS 1.05; PLS three segments, 12.00 long (3.33, 3.94, 4.73).

#### Diagnosis

Males of *Macrothelewuliangensis* sp. n. resemble *M.washanensis* sp. n. by having similar palpal bulb morphology, but they can be distinguished by having spines in prolateral and dorsal views of palpal tibia and similar palpal bulb morphology, females of the new species are similar to others by the apically teardrop shaped receptacula bent inwards apically. Males of *M.wuliangensis* sp. nov. can be distinguished from *M.washanensis* sp. n. having five tibial spines visible in prolateral view, two tibial spines visible in dorsal view (Fig. 8G-I); tibia I has 10 ventral spines with six arranged in three pairs, tibia II has 7 ventral spines (Fig. 9) (vs four tibial spines visible in prolateral view, 0 dorsal spines; tibia I with nine spines visible in ventral view, tibia II has 3 ventral spines in *M.washanensis* sp. n.). Females of *M.wuliangensis* sp. n. can be differentiated from *M.washanensis* sp. n. by the ratio of the length of the T to the length of the CD is almost 1:5 (Fig. 13) (the ratio of the length of the T to the length of the CD is almost 1 : 6 in *M.washanensis* sp. n.).

#### Etymology

The specific name refers to the type locality and is a noun in apposition.

#### Distribution

China, Yunnan Province (Jingdong, Zhenyuan) (Fig. 15)

#### Ecology

**Figure 14.**
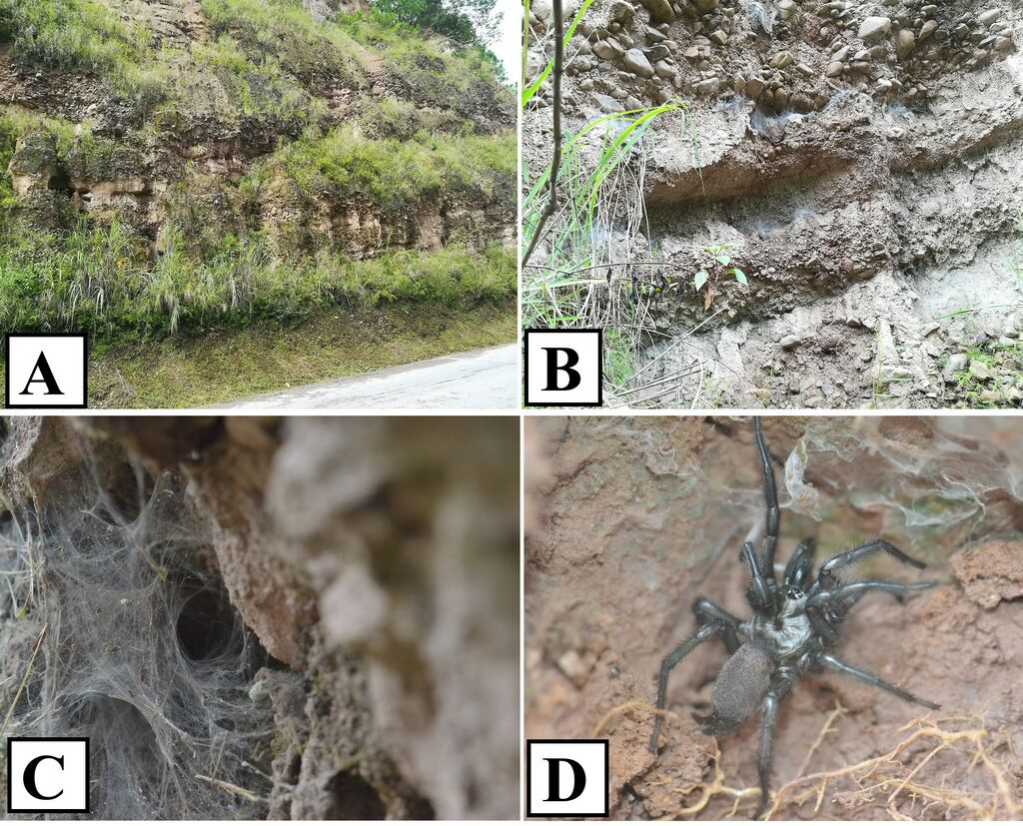
*Macrothelewuliangensis* sp. n. **A** Microhabitat; **B** Spider shelter; **C** Web; **D** Living female.

**Figure 15.**
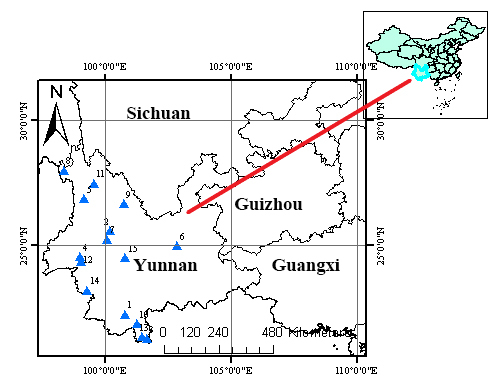
Distribution records of the *Macrothele* in Yunnan Province. **1.** *M.bannaensis* Xu & Yin, 2001; **2.** *M.cangshanensis* Yang et al., 2018; **3.** *M.undata* Tang, Zhao & Yang, 2020; **4.** *M.jinlin* Yang et al., 2018; **5.** *M.jingzhao* Chen, Jiang & Yang, 2020; **6.** *M.multispine* Wang, Li & Yang, 2019; **7.** *M.sanheensis* Tang, Zhao & Yang, 2020 ; **8.** *M.yani* Xu, Yin & Griswold, 2002, Shi et al. 2018; **9.** *M.yongshengensis* Yang, Zhao & Yang, 2019; **10.** *M.menglunensis* Li & Zha, 2013; **11.** *M.yunlingensis* Yang, Zhao & Yang, 2019; **12.** *M.arcuata* Tang, Zhao & Yang, 2020; **13.** *M.yunnanica* Zhu & Song, 2000; **14.** *M.washanensis* sp. n.; **15.** *M.wuliangensis* sp. n.

These spiders usually live in the gap between high rocks and soil (Fig. 14A). They use the natural gap to form a web (Fig. 14B). The web is obvious, funnel shaped, and there are many gaps (Fig. 14C). The spiders usually stay at the hole and wait for the appearance of prey. Generally, the female is large (Fig. 14D).

#### Variation

Male (holotype and two male paratypes, n = 3): total length 22.05-22.63; maxillary cuspules 210-330, labial cuspules 57-79. Female (paratypes, n = 16): total length 19.27-26.86; maxillary cuspules 219-354, labial cuspules 61-84.

## Discussion

Morphologically, the two new species described is different from that of other species. In addition, the interspecific genetic distance was calculated. In Table 1, the intraspecific genetic distance of the *M.wuliangensis* sp. n. is 0.0053, less than 0.02, and the interspecific genetic distance between the *M.wuliangensis* sp. n. and other species is 0.0613-0.5489; The interspecific genetic distance between *M.washanensis* sp. n. and other species is 0.0613-0.5832. The females and males of two new species are collected from the same location, therefore, we consider that the two new species proposed are effective.

## Supplementary Material

XML Treatment for Macrothele washanensis

XML Treatment for Macrothele wuliangensis

## Figures and Tables

**Table 1.** Interspecific genetic distance of two new species based on the p-distance mode.

GenBank number	Species	*M.yani*	*M.wuliangensis* sp. n.	*M.wuliangensis* sp. n.	*M.washanensis* sp. n.	*M.jinlin*
MW868388	*M.yani*					
OP003884	*M.wuliangensis* sp. n.	0.1839				
MW847615	*M.wuliangensis* sp. n.	0.1891	0.0053			
OP484957	*M.washanensis* sp. n.	0.2119	0.0630	0.0613		
MW850365	*M.jinlin*	0.5849	0.5849	0.5814	0.5832	

## References

[B8007365] Chen Hui-Min, Jiang Xuan-Kong, Yang Zi-Zhong (2020). Two new species of the genus *Macrothele* of China (Araneae, Macrothelidae). Journal of Guangxi Normal University (Natural Science Edition).

[B8007091] Hu Jin-Lin, Li Fu-Jiang (1986). On two species of *Macrothele* from China (Araneae: Dipluridae). Acta Zootaxonomica Sinica.

[B8007405] Lin Ye-Jie, Yan Xun-You, Li Shu- Qiang, Ballarin F, Chen Hai -Feng (2021). Five new species of *Macrothele* Ausserer, 1871 from China (Araneae, Macrothelidae). ZooKeys.

[B8007082] Li Shu-Qiang, Zha Zu-Wei (2013). *Macrothele* spiders from Xishuangbanna rainforest of Yunnan, China (Araneae, Hexathelidae). Acta Zootaxonomica Sinica.

[B8007100] Pocock R. I. (1901). On some new trap-door spiders from China. Proceedings of the ZoologicalSociety of London.

[B8007073] Saitô S (1933). Notes on the spiders from Formosa. Transactions of the Sapporo Natural History Society.

[B8007321] Shi Ji-Hui, Yang Zhi-Bin, Zhao Yu, Yang Zi-Zhong (2018). The first description on the male of *Macrotheleyani*, and supplemental descriptions of female (Araneae: Macrothelidae). Acta Arachnologica Sinica.

[B8007064] Shimojana M, Haupt J (1998). Taxonomy and natural history of the funnel-web spider genus *Macrothele* (Araneae: Hexathelidae: Macrothelinae) in the Ryukyu Islands (Japan) and Taiwan. Species Diversity.

[B8006882] Simon E (1892). Histoire naturelle des araignées.

[B8007223] Song Da-Xiang, Zhu Ming-Sheng, Chen Jun (1999). The spiders of China.

[B8007387] Tang Ya-Ni, Zhao Yu, Yang Zi-Zhong (2020). Three new species of the funnel-web spider genus *Macrothele* from the Southwest China (Mygalomorphae: Macrothelidae). Zootaxa.

[B8007785] Tang Ya-Ni, Wu Ya-Ying, Zhao Yu, Yang Zi-Zhong (2022). Description of a new genus and two new species of the funnel-web mygalomorph (Araneae: Mygalomorphae: Macrothelidae) from China with notes on taxonomic amendments. Zootaxa.

[B8007356] Wang Ying, Li Yue, Yang Zi-Zhong (2019). A new species of genus *Macrothele* (Araneae: Macrothelidae) from China. Acta Arachnologica Sinica.

[B8007056] Catalog World Spider World Spider Catalog. Version 23.5. Natural History Museum Bern. http://wsc.nmbe.ch.

[B8007303] Xu Xiang, Yin Chang-Ming (2001). A new species of the genus *Macrothele* from China (Araneae: Hexathelidae). Journal of Natural Science of Hunan Normal University.

[B8007167] Xu Xiang, Yin Chang-Ming, Griswold C. E (2002). A new species of the spider genus *Macrothele* from the Gaoligong Mountains, Yunnan, China (Araneae: Hexathelidae). Pan-Pacific Entomologist.

[B8007338] Yang Zhi-Bin, Zhao Yu, Zhang Cheng-Gui, Yang Zi-Zhong (2018). Two new species of the genus *Macrothele* from the southwest of China (Mygalomorphae: Macrothelidae). Acta Arachnologica Sinica.

[B8007347] Yang Zhi-Bin, Zhao Yu, Yang Zi-Zhong (2019). Two new species of the genus *Macrothele* from the southwest of China (Mygalomorphae: Macrothelidae). Journal of Dali University.

[B8008310] Zhu Ming-Sheng, Song Da-Xiang (2000). Review of the Chinese funnel-web spiders of the genus *Macrothele*, with descriptions of two new species (Araneae: Hexathelidae). Raffles Bulletin of Zoology.

[B8007285] Zhu Ming-Sheng, Li Ting-Hui, Song Da-Xiang (2000). A new species of the genus *Macrothele* (Araneae: Hexathelidae) from China. Journal of Hubei University.

